# Burn Injury Due to Cyanoacrylate-Based Nail Glue: A Case Report and Literature Review

**DOI:** 10.7759/cureus.13878

**Published:** 2021-03-14

**Authors:** Taghreed R Alhumsi, Qutaiba N Shah Mardan

**Affiliations:** 1 Division of Plastic Surgery, Department of Surgery, King Saud University Medical City, Riyadh, SAU; 2 Plastic and Reconstructive Surgery Section, Surgery Department, King Faisal Specialist Hospital and Research Centre, Riyadh, SAU

**Keywords:** burn, thermal, chemical, cyanoacrylate, case report

## Abstract

Cyanoacrylate is used in the manufacturing of surgical adhesives, nail glue, and super glue. After contact with cotton or wool, among other catalysts, an exothermic reaction is ignited, inflicting thermal damage to the skin underlying clothes. Nine papers have been published about such an incidence, majority of which involve children. This is the first paper that presents the case of such a burn condition in Saudi Arabia. A four-year-old girl suffered a mix of first- and second-degree burns due to cyanoacrylate spillage over the digits, anterior aspect of the right thigh, and a patch of the skin on the left upper abdomen, spanning 4% of the total body surface area. The clothes were forcefully removed by the mother, and shampoo was applied over the area. At the emergency department, irrigation with normal saline was performed followed by dressing with paraffin-impregnated gauzes and silver sulfadiazine. Volar slabs were placed on the hands. The dressing in the hands was later changed to fusidic acid as the burn healed. Silicone sheet dressing was initiated 28 days later after the burned skin had healed. At one-year follow-up, the wounds were fully healed with no abnormal scar formation. This paper aims to improve awareness about proper first-aid burn management, which determines the quality of the outcome. Further emphasis is required on providing a safe environment for the children and clear, comprehensible warning of hazards on the label of the cyanoacrylate based-products.

## Introduction

Many beauty products are used daily and are deemed safe for utility, such as cyanoacrylate glue. Categorized as an organic monomer, cyanoacrylate glue is consumed as super glue, tissue adhesive, and attachment of artificial nails [1,2]. It has found a remarkable fame during the Vietnam War as an adhesive for the closure of organs and skin injuries. However, reports of toxicity had dampened its use [2]. Different forms such as methyl 2-cyanoacrylate and ethyl 2-cyanoacrylate are available as super glue [3]. Butyl-2-cyanoacrylate until recently was the only commercially available form; 2-octyl-cyanoacrylate was approved by the US FDA in 1998 as a tissue adhesive and for skin closure due to the lower toxicity and skin reaction [1]. We present a case of a child who suffered from burns due to spillage of nail glue over her clothes and was managed in a tertiary care university hospital. Only nine reports have been published in the literature about cyanoacrylate-mediated burns till the writing of this paper [1-9]. We aim to raise awareness about proper first-aid management and emphasize the importance of providing a hazard-free environment for children.

This article was previously presented as a poster at the 2nd International Society of Aesthetic Plastic Surgery Meeting in Riyadh, Kingdom of Saudi Arabia, December 8, 2019.

## Case presentation

**Figure 1:**
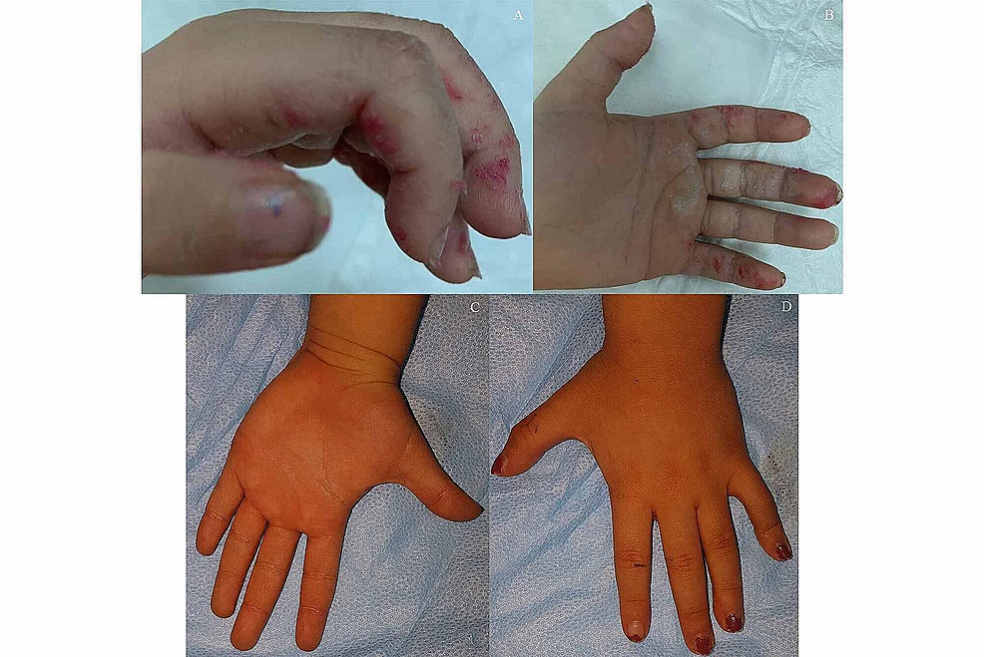
Thermal injury inflicted to the digits (A and B) Injury sustained to the digits. The pictures were taken in the emergency department. (C and D) The condition of the hand 45 days following the accident. The burn has healed uninterruptedly.

**Figure 2:**
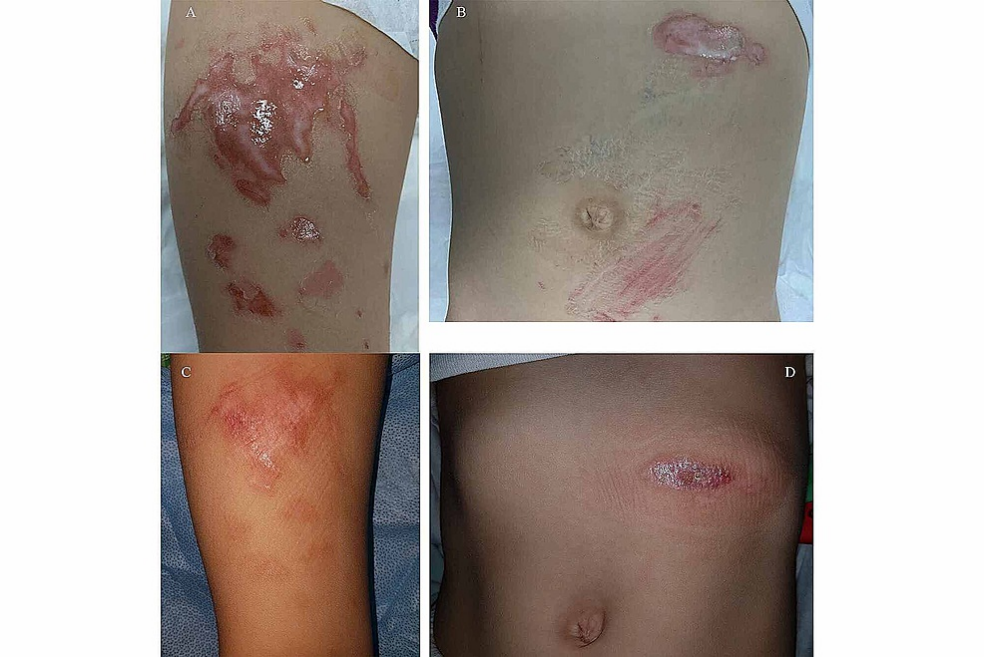
Thermal injury inflected on the abdomen and right thigh (A and B) Injury sustained to the middle anterior aspect of the right thigh and the upper left abdomen and near the umbilicus, respectively, taken in the emergency department. (C and D) Images taken 45 days after the accident. Both of the affected areas have healed well.

**Figure 3:**
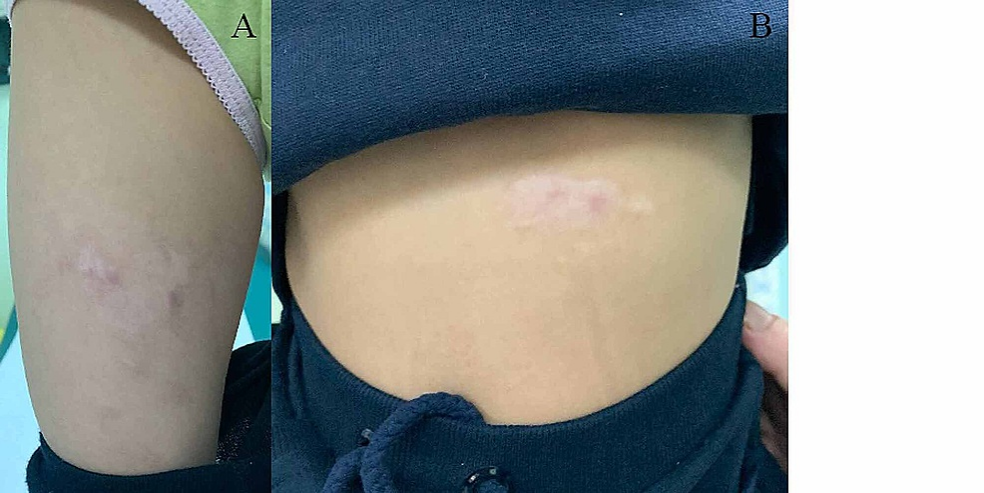
Scars of the thermal injury to the abdomen and right thigh on one-year follow-up (A and B) Injury sustained to the middle anterior aspect of the right thigh and the upper left abdomen and near the umbilicus, respectively. Images were taken one year after the injury and show complete healing.

A four-year-old girl, not known to have any medical illnesses, arrived at the emergency department (ED) after she accidentally spilled nail glue over her abdomen, right thigh, and both hands. The mother promptly removed her cotton clothes, which were stuck to the body, and applied shampoo over the involved area. No irrigation was performed at home. In the ED, she was assessed and examined. Her vital signs were stable. A total body surface area of approximately 4% was affected by first- to second-degree burns. The involved areas were (a) patches of the volar and dorsal aspect of all fingers proximally reaching the metacarpophalangeal joints, (b) a 5 x 3.5 cm area of the middle anterior aspect of the right thigh, and (c) a patch of 2 x 5 cm affecting the upper left aspect of the abdomen and erythematous area near the left lower aspect of the umbilicus. No blisters were found. Irrigation using 4 liters of warm normal saline was performed, and the affected areas were dressed using paraffin-impregnated gauze over a layer of silver sulfadiazine. Volar slabs were applied for both hands. The child was admitted for 24 hours for observation and analgesia. Both hands were kept elevated and frequently assessed for capillary refill. The patient was then followed up by the plastic surgery team weekly in the dressing clinic. Three days following the incidence, silver sulfadiazine cream was substituted with topical fusidic acid 2% as the injury showed good healing. After 28 days, the burned areas were healed and silicone sheet dressing was started. At one-year follow-up, the wound was completely healed without hypertrophic scarring (Figures 1-3).

## Discussion

Sixteen years after its discovery in 1942, cyanoacrylate was widely available for domestic use. It is composed of a cyano group and an ester formed by an acryl acid, alcohol, and a double-bonded central carbon atom. The monomers solidify after rapid polymerization when mixed with weak alkaline material via an exothermic reaction. Many factors catalyze this process, where the double bond is broken, such as chemicals, electromagnetic radiation, and, more relevant to this case report, cotton and wool. Sufficient amount of hydroxyl groups for the polymerization reaction is provided by the β-linked glucose units that form cotton, and due to its catalytic effect, heat accumulates rapidly with even a small amount of fabric - a process that culminates into spontaneous ignition of the clothes inflicting damage to the underlying skin [2].

The released white smoke may trigger asthma attacks [3]. Factors increasing the intensity of the burn include a larger volume of the substance, lower viscosity that facilitates substance distribution leading to a larger burn surface area, and the type of cyanoacrylate product, particularly those with shorter alcohol sidechains [2]. Besides cotton, water and bases can facilitate the exothermic reaction. A young lady sustained full-thickness burn after spillage of nail glue over her denim jeans. It is postulated that sweat provided a moisture environment, while the remnants of the washing agent, a weak base, have aggravated this reaction [8].

**Figure 4:**
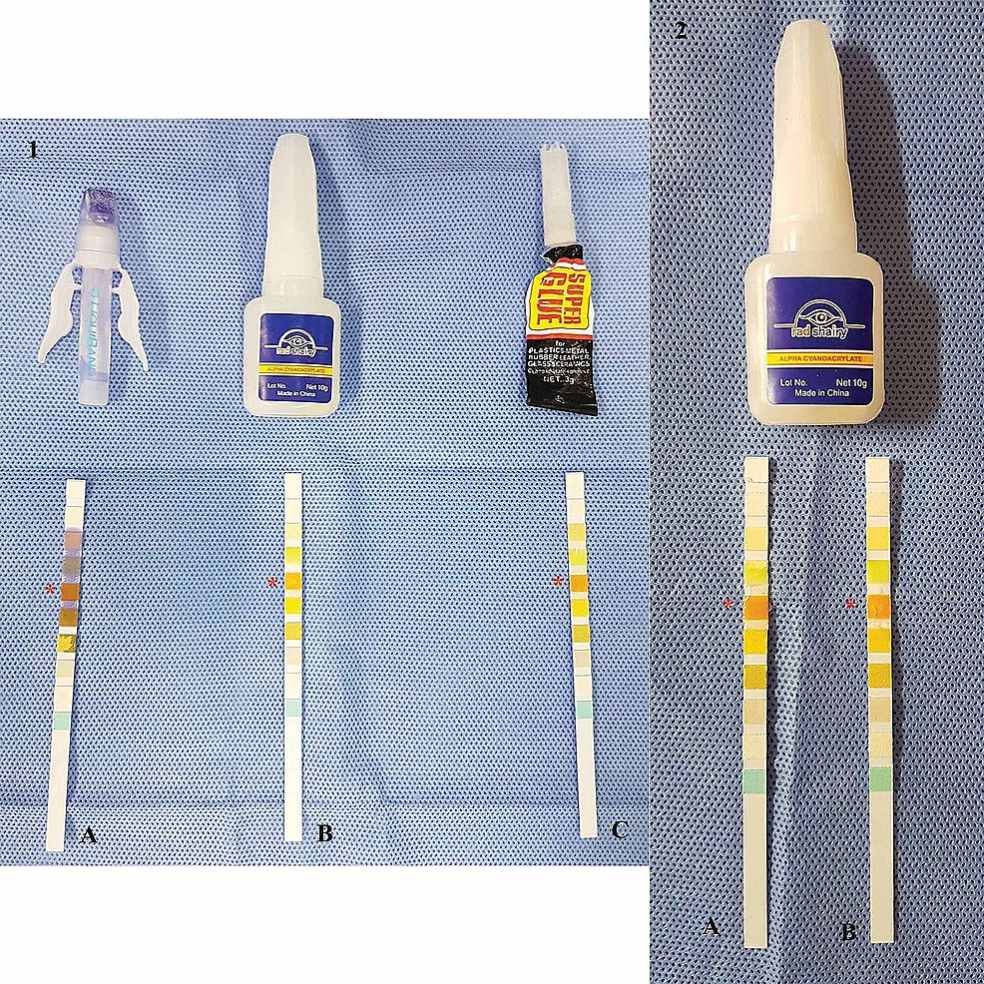
Testing the cyanoacrylate pH with dip-sticks Picture 1 shows the three different forms of cyanoacrylate along with the dip-stick used to measure their pH. There is no significant change in pH or difference between the three components. Referring back to the bottle containing the sticks, the pH would be below 5. The bluish discoloration on stick A is due to the surgical glue. (A) Surgical glue. (B) Nail glue. (C) Super glue. (*) The box for pH measurement. Picture 2 is taken after adding shampoo to a new stick immersed in the nail glue. There is no difference in pH between cyanoacrylate alone or when shampoo is added. (A) Cyanoacrylate with shampoo. (B) Cyanoacrylate alone. (*) The box for pH measurement.

In an effort to further study the changes accompanying cyanoacrylate application, the authors conducted an experiment using urinary dip-sticks to note any pH changes. Three different cyanoacrylate compounds were used: the same nail glue used by the family, super glue, and a surgical adhesive. After the application of each compound on a separate dip-stick for a minute, there was no alteration in the color, implying that no pH change was noted. To simulate the conditions of this case report, we added a small amount of shampoo to a dip-stick coated with the nail glue. However, no change was noted (Figure 4).

Optimal management should follow the standard of care for thermal burns. Clothing stuck to the affected area should not be forcefully removed to avoid the shearing effect on the underlying skin, which results in more pain and skin damage [1,2]. In addition to warm water irrigation, adherent clothes can be removed using petroleum gel, saline, or 5% sodium bicarbonate solution. It is inadvisable to apply acetone over broken skin, orifices, and mucosa due to the discomfort and pain. When used over intact skin, it should be followed by moisturizing cream application to prevent dryness [2]. Cotton swabs should never be used as they may ignite a violent reaction with the glue. Once the cyanoacrylate has dried over the skin, nail emery board or pumice stone soaked in warmed water can be used to remove the glue [1].

Table 1 summarizes the reported cases in nine published papers on cyanoacrylate victims. Nine out of 15 cases in the literature involved victims aged 12 years and younger. Five cases were of children younger than two years. Overall, pediatric burns pose an ongoing challenge for plastic surgeons, and further effort is required to improve parent education regarding safety of home surroundings for children both globally and nationally [5,10]. Majority of cases, including the patient in this report, healed without complications with good care except for three cases that developed a hypertrophic scar [6] or infections [9].

**Table 1:** Summary of the reports describing cases of cyanoacrylate-induced burns ER, emergency room; STSG, split-thickness skin grafting

Paper	Patient	Accident	Intervention	Prognosis
Ting et al [8]	A 10-year-old boy	Partial-thickness burn of the fingers of both hands	Irrigation at home; liquid paraffin to remove the adherent substance; lignocaine gel for analgesia	No data are provided
Hakan et al. [6]	A 5-year-old girl	Full-thickness burn over the left thigh	Prompt clothing removal and irrigation; family refused grafting; managed with dressing	Healed after 23 days leaving a hypertrophic scar
Eyth et al. [2]	A 2-year-old boy	Mixed-depth partial-thickness burn to the face, chest, and abdomen	Inappropriate first-aid measures; mesh and nitrofural dressing	Healed within 28 days
	A 13-year-old girl	Mixed-depth partial-thickness burn to the foot dorsum	Irrigation and coverage with cling-film; mesh and ACTICOAT dressing with splint immobilization of the foot	Healed within 21 days
Coles et al. [5]	A 15-year-old girl	Mixed-depth burn to the upper medial right thigh	No first aid at home; irrigation, dressing, and antibiotics at ER; referred to burn center; then Mepilex^®^ Ag and flamazine dressing was applied and finally covered with STSG	Complete healing after two weeks from grafting
	A 3.5-year-old girl	Burn to the dorsum of the right forearm and hand	Immediate clothing removal and irrigation. Mepilex^®^ Lite applied	Healed; no further data are provided
	A 1.5-year-old girl	Full-thickness burn to the right inner thigh after accidental nail glue spillage, was wearing pajamas	Immediate clothing removal with irrigation; treated with dressing due to small affected area	Healed after five weeks
	A 2-year-old boy	Burn to the left hand, abdomen, thigh, and knee	Immediate clothing removal and irrigation; discharged as no burn was identified on examination	No data are provided
Kelemen et al. [9]	A 15-year-old girl	Full-thickness burn to the right anterior thigh	Irrigation and anti-septic cream at home; excision of skin and STSG	Postoperative infection treated with antibiotics; uncomplicated healing after three months
	An 11-year-old girl	Full-thickness burn to both inner thighs	No first-aid; tangential skin excision followed by STSG and pressure garment	Complete healing after three months
	A 16-year-old girl	Full-thickness burn on the left lower leg	No first-aid; tangential skin excision followed by STSG and pressure garment	Postoperative infection treated with antibiotics; uncomplicated healing after three months
Bélanger et al. [4]	A 5-month-old girl	Second-degree burn to the abdomen	No first-aid; hospitalization for treatment and supervision; flamazine dressing	No data are provided
Clarke [1]	A 2-year-old boy	Full-thickness burn to the right lower posteromedial aspect of the leg	Partial-thickness developed to full thickness due to forceful clothing removal; tangential excision of burned area followed by STSG; Mepitel^®^ and pressure dressing	Full graft intake after two days; however, no data are provided about later results
Jamnadas-Khoda et al. [3]	An 82-year-old gentleman	Full-thickness burn to the left medial thigh	Irrigation was performed; STSG	Full graft up-take at three months
Tang et al. [7]	A 28-year-old lady	Full-thickness burn to the upper medial thigh	Unnoticed initially by the patient; the culture, few days later, grew pseudomonas; no improvement with antibiotics; surgical debridement and STSG	Full graft up-take on subsequent visits; no further data are provided

In this case report, the product spilled, according to the label, was a mixture of water with ethyl cyanoacrylate and polymethyl methacrylate. The thermal reaction was most likely expedited by the clothes and the shampoo applied by the mother attempting to relieve the burn. Hence, the heat produced by the cyanoacrylate was increased, causing the burns. When reviewing the product label, it was found that it advised keeping the product far from fabric as it may ignite thermal reaction. Also, relevant precautions were provided in case the glue was spilled over the eyes. However, the warning was written in English, which is not spoken by a significant portion of the public in Saudi Arabia.​​​​​​​

## Conclusions

The benefits of cyanoacrylate-based products make them easily accessible for domestic use. However, the harm they may inflict is considerable, ranging from irritating chronic dermatitis to a devastating burn that may necessitate surgical intervention. Immediate and proper management, through following the standard care of thermal injury, limits further damage. But the most important factor in the management of this injury is by prevention. This case report highlights the importance of a safe environment around children, the main affected population by this product. In addition, the warning labels on these hazardous products should be simple, clear, and in the language spoken by the local population. No significant change in pH was noted between the different types of cyanoacrylate even after applying shampoo over the nail glue.

## References

[1] Clarke TFE (2011). Cyanoacrylate glue burn in a child--lessons to be learned. J Plast Reconstr Aesthetic Surg.

[2] Eyth CP, Echlin K, Jones I (2019). Cyanoacrylate burn injuries: two unusual cases and a review of the literature. Wounds Res.

[3] Jamnadas-Khoda B, Khan MAA, Thomas GPL, Ghosh SJ (2011). Histoacryl glue: a burning issue. Burns.

[4] Bélanger RE, Marcotte ME, Bégin F (2013). Burns and beauty nails. Paediatr Child Health.

[5] Coles C, Javed MU, Hemington Gorse S, Nguyen D (2016). Paediatric burns secondary to nail adhesives: a case series. Burns Trauma.

[6] Hakan A, Tarıkçı Kılıç K, Kaçar CK (2017). Accidental full thickness burns by super glue. Ann Med Health Sci Res.

[7] Tang CL, Larkin G, Kumiponjera D, Rao GS (2006). Vanity burns: an unusual case of chemical burn caused by nail glue. Burns.

[8] Ting CD, Rosman R, Rahmat R (2018). PP043 Superglue thermal burn. Malays J Emerg Med.

[9] Kelemen N, Karagergou E, Jones SL, Morritt AN (2016). Full thickness burns caused by cyanoacrylate nail glue: a case series. Burns.

[10] Alsalman AK, Algadiem EA, Alalwan MA, Farag TS (2015). Epidemiology of infant burn in Eastern Saudi Arabia. Saudi Med J.

